# Hindlimb Motion during Steady Flight of the Lesser Dog-Faced Fruit Bat, *Cynopterus brachyotis*

**DOI:** 10.1371/journal.pone.0098093

**Published:** 2014-05-23

**Authors:** Jorn A. Cheney, Daniel Ton, Nicolai Konow, Daniel K. Riskin, Kenneth S. Breuer, Sharon M. Swartz

**Affiliations:** 1 Department of Ecology and Evolutionary Biology, Brown University, Providence, Rhode Island, United States of America; 2 School of Engineering, Brown University, Providence, Rhode Island, United States of America; University of Zurich, Switzerland

## Abstract

In bats, the wing membrane is anchored not only to the body and forelimb, but also to the hindlimb. This attachment configuration gives bats the potential to modulate wing shape by moving the hindlimb, such as by joint movement at the hip or knee. Such movements could modulate lift, drag, or the pitching moment. In this study we address: 1) how the ankle translates through space during the wingbeat cycle; 2) whether amplitude of ankle motion is dependent upon flight speed; 3) how tension in the wing membrane pulls the ankle; and 4) whether wing membrane tension is responsible for driving ankle motion. We flew five individuals of the lesser dog-faced fruit bat, *Cynopterus brachyotis* (Family: Pteropodidae), in a wind tunnel and documented kinematics of the forelimb, hip, ankle, and trailing edge of the wing membrane. Based on kinematic analysis of hindlimb and forelimb movements, we found that: 1) during downstroke, the ankle moved ventrally and during upstroke the ankle moved dorsally; 2) there was considerable variation in amplitude of ankle motion, but amplitude did not correlate significantly with flight speed; 3) during downstroke, tension generated by the wing membrane acted to pull the ankle dorsally, and during upstroke, the wing membrane pulled laterally when taut and dorsally when relatively slack; and 4) wing membrane tension generally opposed dorsoventral ankle motion. We conclude that during forward flight in *C. brachyotis*, wing membrane tension does not power hindlimb motion; instead, we propose that hindlimb movements arise from muscle activity and/or inertial effects.

## Introduction

Bats are well known for their forelimbs, which are significantly modified into wings, but their hindlimbs are modified as well. The femur and tibia are more slender than those of comparably-sized non-bat mammals [1], [2], [3], and, in addition, the wing membrane is anchored to the ankle. As a consequence, hindlimb movements contribute to three-dimensional wing shape. Potential aerodynamic consequences of wing shape alteration by these movements may include modulation of lift, drag, and pitching moment. However, the extent of such motion during bat flight is rarely documented, and it is not known whether hindlimb movements during flight are actively controlled or result from external forces acting on the limb.

**Figure 1:**
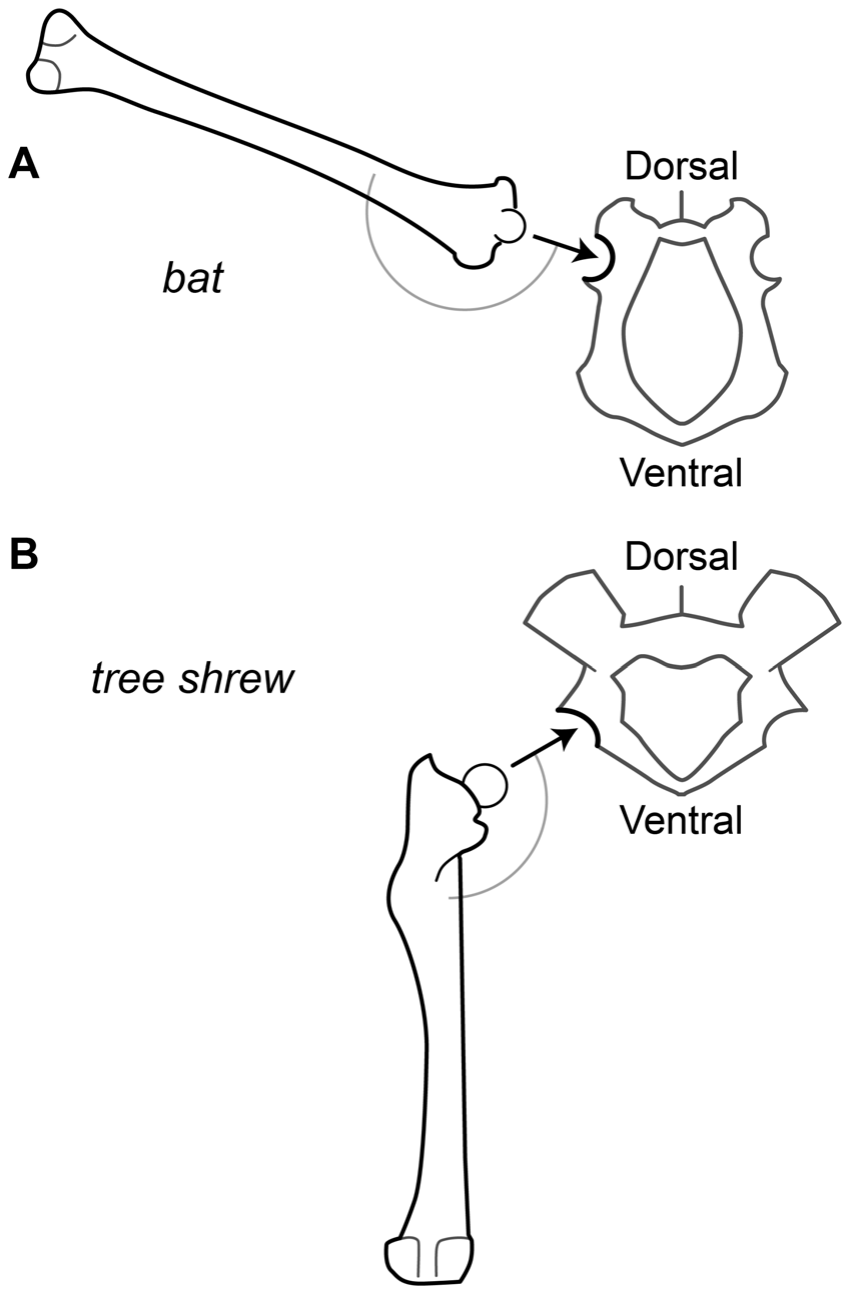
Modifications to the bat hindlimb skeleton. Posterior view of the pelvis and left femur in (A) a bat and (B) a tree shrew, illustrative of the ancestral condition for bats. The arrow indicates the orientation of the acetabulum. The arc indicates the angle between the acetabulum and the shaft of the femur. Modified from [4].

**Figure 2:**
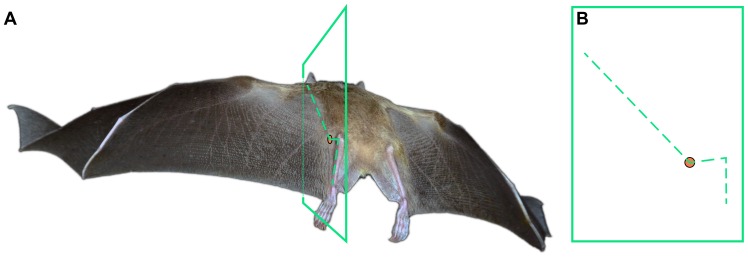
The hindlimb serves as a boundary condition for the wing membrane. (A) Posterior view of *Rousettus aegyptiacus* (Family: Pteropodidae) in flight; flight kinematics in this species are similar to those of *Cynopterus brachyotis* [14]. A plane transects the wing membrane adjacent to the body, femur, and tibia. The wing profile approximates the geometry of these elements where they directly support the skin. (B) A perspective-corrected wing profile view. The orange circle indicates the left hip location. Photo courtesy of Brock Fenton.

Evolutionary reorganization of bat hip anatomy may provide clues to the importance of hindlimb movement during bat flight. The bat hindlimb is rotated 90° or more relative to the ancestral mammal condition (Fig. 1). The ischium is tilted dorsally, orienting the face of the acetabulum dorsally and laterally, and the femoral head and shaft lie along approximately the same axis [4]. Consequently, the knee is oriented dorsolaterally and its flexion moves the ankle and foot ventrally, rather than dorsally, the basal condition for mammals (Fig. 2). Thus, knee flexion pulls the trailing edge of the wing ventrally rather than dorsally.

Because the wing is attached to the hindlimb, dorsoventral movement of the hindlimb affects aerodynamics by modifying wing shape and angle of attack (Fig. 2). This enables bats to control the trailing edge of the wing in a manner similar to modern aircraft. Mechanical models of bats with fixed wings have demonstrated that flexion of the hindlimb can considerably increase both lift and the lift-to-drag ratio [5]. However, neither the forelimbs nor the hindlimbs remain stationary during bat flight [6]-[11], so understanding the dynamics of limb motion is integral to interpreting the aerodynamic effects of hindlimb position.

Bat hindlimbs are known to move both dorsoventrally and mediolaterally during flight [7], [9], [10]. During downstroke, dorsoventral hindlimb motion that is in phase with the wingbeat cycle increases angle of attack of the proximal wing, while hindlimb motion that is out of phase with the wingbeat cycle reduces this effect. During take-off, vespertilionid bats oscillate their hindlimbs, tail, and uropatagium through a large dorsoventral arc, and the motion of the hindlimb and forelimb begin in phase, and then move out of phase by as much as 180 degrees [10]. During steady flight, the hindlimbs of *Glossophaga soricina* (Family: Phyllostomidae) move up and down in phase with the wings, and it has been suggested that the amplitude of the motion may be more pronounced at low flight speeds [7].

Here, we examine the movement of the hindlimbs and trailing edge of the wing membrane during flight in the lesser dog-faced fruit bat, *Cynopterus brachyotis* (Family: Pteropodidae), and discuss potential aerodynamic consequences of these movements. In many species, hindlimb movements will influence both the wing membrane and the tail membrane. We are able to isolate hindlimb influence on the wing in *C. brachyotis* because it has a significantly reduced tail membrane and lacks a tail. We propose that hindlimb motion could arise from at least three sources: 11) passive tension exerted by the wing membrane may cause hindlimb motion; 22) hip and knee muscles may directly power hindlimb movement; and/or 33) vertical body oscillations could impose oscillations on the hindlimb via inertial effects. We provide a body-referenced kinematic description of ankle translation, which occurs in response to rotations at the hip and/or knee, and using this data we address the first of these possibilities. Accordingly, our aims are to: i) describe ankle motion over the course of a wingbeat cycle, and ii) describe the orientation and length of the wing membrane trailing edge over the course of a wingbeat cycle. From these data, we test the hypotheses that 1) ankle motion amplitude is dependent upon flight speed as previously proposed [7], and 2) that passive wing membrane tension is sufficient to explain the ankle motion we observe.

## Methods

### Bats

Study subjects were adult, not aged, captive-bred female lesser dog-faced fruit bats, *Cynopterus brachyotis* (*N* = 5; mean ± S.D. body mass  = 33.6±4.5 g.) on loan from the Lubee Bat Conservancy (Gainesville, FL, USA). Animals were housed in the animal care facilities of the Harvard University Concord Field Station (Bedford, MA, USA), and provided with food and water *ad libitum*. All experiments complied with ethical treatment policies and protocols, and were authorized by the Institutional Animal Care and Use Committees of Brown University (#67-07), Harvard University (#27-10), the Lubee Bat Conservancy (#CP07-2), and the Division of Biomedical Research and Regulatory Compliance of the Office of the Surgeon General of the United States Air Force (#6F050).

### Kinematic recordings

We recorded the flight kinematics of each individual at a range of speeds. To vary flight speed and to restrict filming volume, we flew each bat in a wind tunnel with test section dimensions of 1.4×1.2×1.2 m (Harvard University Concord Field Station Wind Tunnel). Wind tunnel performance and design parameters have been detailed elsewhere [12]. The flights were recorded at 1000 frames per second using three phase-locked high-speed cameras with resolution of 1024×1024 pixels (Fastcam 1024 PCI; Photron USA, Inc., San Diego, CA, USA). Further details about data collection were reported previously [13].

**Figure 3:**
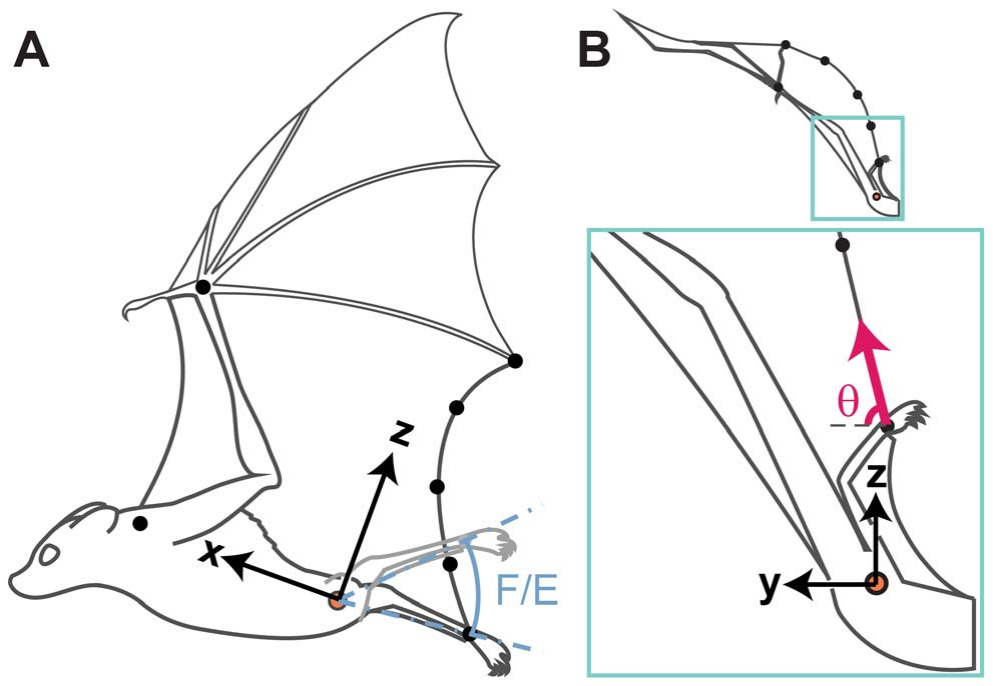
Illustration of the eight anatomical markers used in this study and the parameters measured. The orange circle indicates the left hip marker location. (A) Lateral view from late upstroke. F/E (blue) indicates the amplitude of flexion/extension of the ankle marker in the parasagittal (xz-) plane. (B) Posterior view from early downstroke. Inset, below: magnified view of the wing membrane trailing edge attaching at the ankle. θ (magenta) indicates the angle of the trailing edge at the ankle relative to the y-axis, in the transverse (yz-) plane.

Before experiments, each bat was anesthetized with isoflurane gas, induced at 2.5% and maintained at 2% concentration. The duration of anesthesia was brief, just long enough to place a series of eight markers on the body and the skin of the left wing and hindlimb using a non-toxic acrylic paint marker (Fig. 3A). We recorded flights at nine different speeds for each individual. The flights were brief, lasting a few seconds. As the bat flew through our filming volume, we filmed two to four complete wingbeats. Following each successful trial, the bat rested for at least five minutes. Of the hundreds of wingbeats filmed, we carefully selected twenty-five wingbeats for digitization that best represented straight, steady, symmetrical, uninterrupted flight and captured a broad range of flight speeds for each individual. Based on the three camera views, we selected wingbeats that best exemplified station-holding, in which the body was aligned with the wind tunnel and in which wingbeat kinematics were consistent with those of previous wingbeats and exhibited left-right symmetry in amplitude (dataset provided in File S1). For additional details concerning wingbeat cycle selection and summary statistics, see [14]. We used the upper reversal point of the wrist marker to denote the beginning and end of the wingbeat cycle. The wingbeats used in our study were performed at flight speeds ranging from 3.2 to 7.8 m/s.

To accurately reconstruct marker motion in the filmed wingbeats, the filmed volume of the wind tunnel was calibrated using the direct linear transformation (DLT) method [15]. The calibration was performed using a 35×35×28 cm calibration frame. In cases where a marker was obscured in two or more camera views, the gap in kinematic data was filled using a custom curve-fitting algorithm [13].

### Coordinate system

Reconstructed kinematic data were transformed to a right-handed orthogonal body-referenced linear coordinate system, with the origin at the hip marker. The orientation of the x-axis was aligned with the shoulder and hip, the y-axis was made orthogonal to the x-axis and gravity (positive  =  bat's left), and the z-axis was made orthogonal to the x-axis and y-axis (positive  =  dorsal) (Fig. 3). The anatomical planes that can be approximated by pairs of axes are parasagittal (xz-), frontal (xy-), and transverse (yz-) planes. We marked and digitized only the left wing because cameras were focused on a single side of the body. By focusing on the left wing and body, we increased spatial resolution by reducing filming volume, and increased temporal resolution by minimizing marker occlusion with careful camera placement. We then carefully selected straight, unperturbed flight trials for analysis because we did not measure roll of the bat, and because adding roll as an additional degree of freedom of motion would have added greater complexity to the analysis.

### Ankle marker kinematics and statistics

We describe ankle motion relative to the hip in the body-referenced coordinate system. We project the vector from hip to ankle onto the frontal (xy-) and parasagittal (xz-) planes. The projected rotations about the hip, relative to the x-axis, are described as abduction/adduction and flexion/extension respectively. These terms are typically reserved for rotations about a single joint, but our usage describes the combined rotations of the hip and knee joints. The difference between the maximum and minimum values observed within a wingbeat was designated as the amplitude of motion (Fig. 3). We then tested for speed-dependent change in abduction/adduction and flexion/extension amplitude. Prior to statistical tests, we performed outlier analysis, and then conducted mixed-model regression, testing for speed-dependent change in each amplitude. To account for variation among individuals, we included individual as a random effect (Systat 12, Systat Software, Inc.).

### Trailing edge length and orientation

To approximate the force exerted on the ankle by the wing membrane in the transverse (yz-) plane, we calculated trailing edge length as a proxy for the magnitude of tension in the wing membrane, and the tangent of trailing edge shape at the ankle, as a proxy for the direction of that tensile force. As the wing membrane elongates elastically, tension is generated internally throughout the membrane, which is ultimately transmitted to supporting skeletal elements. For elastic deformations, the wing membrane behaves in a spring-like manner, requiring greater force to produce greater elongation. To calculate the trailing edge length and shape, we fit a parabola to the locations of the ankle, the tip of digit V, and three markers spaced evenly between these skeletal landmarks along the trailing edge. We selected a parabola because we found that it provided a good fit to our five markers. Seven wingbeats in which it was not possible to digitize all trailing edge markers were excluded from this portion of the analysis.

We calculated trailing edge length by integrating the arc length of the parabola. To compare across individuals, we normalized length by the maximum observed within a wingbeat. We found that maximum length was consistent within an individual, and the timing of maximum length with respect to the wingbeat cycle was consistent among individuals. Ideally, wing membrane length in an unloaded configuration would be our reference length, however, this measurement was less consistent both within and among individuals. We suspect this may be in part due to the J-shaped stress-strain behavior of the wing membrane [16], where perturbations in aerodynamic load will produce very different changes in membrane length depending on whether the membrane is loaded versus unloaded when the perturbation is applied. When loaded, the wing is relatively stiff and perturbations cause small changes in length, but when the wing is relatively unloaded, and therefore quite compliant, the same magnitude of force perturbation will cause a much greater change in length. Thus, under stochastic forcing, wing membrane length would vary less at high strains.

The orientation of the wing membrane trailing edge, where it attaches to the ankle, was used to estimate the orientation of tension acting on the ankle in two dimensions. We could not resolve the rostrocaudal (x) component of force, so this component was omitted from our analysis. We described the direction of tension on the ankle in the transverse (yz-) plane by calculating the angle, θ, formed by the trailing edge relative to the mediolateral (y-) axis (Fig. 3B).

### Normalization of the wingbeat time-course

To compare multiple wingbeats, the time course of all trials was normalized to percent wingbeat cycle. Transitions from upstroke to downstroke were defined by the minimum and maximum dorsal position of the wrist. We defined downstroke as comprising 0 to 50 percent and upstroke as 50 to 100 percent of the wingbeat cycle. Empirically, downstroke occupied 48% of the wingbeat cycle on average.

### Wing membrane-opposed hindlimb motion

When ankle velocity is oriented opposite to the direction of wing membrane tension on the ankle, then the wing membrane removes power from ankle motion (motions of the ankle help drive wing membrane motion or deformation). Conversely, when ankle velocity and wing membrane tension are aligned, the wing membrane adds power to ankle motion (motions of the membrane help drive ankle movement). To estimate whether the wing membrane aided or opposed dorsoventral ankle motion, we calculated the percentage of time that the dorsoventral (z) component of ankle velocity and membrane tension, sin(θ), were opposed (i.e., one was positive, while the other was negative). We did not consider the mediolateral (y) component because we found that membrane tension exerted significant lateral, but not medial, force on the ankle, which is likely due to an anatomical constraint (Fig. 2).

## Results

### Effect of speed and individual on the amplitude of ankle marker motion

Prior to carrying out statistical analysis of the relationship among speed, amplitude, and individual, we removed one outlier from the measurements of flexion/extension motion. We found no effect of speed, individual, or their interaction on the flexion/extension amplitude of ankle motion (ANOVA; speed: F_1,14_ = 1.859; p = 0.194; individual: F_4,14_ = 0.732; p = 0.211; interaction-term (speed × individual): F_4,14_ = 0.645; p = 0.640) or on the abduction/adduction amplitude of ankle motion (speed; F_1,15_ = 0.505; p = 0.488; individual: F_4,15_ = 0.653; p = 0.634; speed × individual: F_4,15_ = 0.664; p = 0.627). The results of our analysis did not change significantly when the outlier was included.

### Ankle marker kinematics

**Figure 4:**
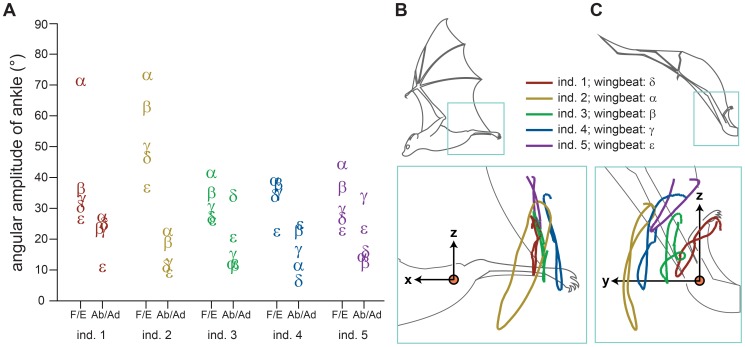
Ankle position was variable among wingbeats. Variation in ankle motion amplitude and path. Letters α-ε each represent a separate wingbeat. The wingbeats are assigned alphabetically from greatest to smallest dorsoventral amplitude. (A) Angular amplitude of the ankle relative to the hip for flexion/extension (F/E) and abduction/adduction (Ab/Ad), separated by individual. A given letter within an individual is for a single wingbeat, across the F/E and Ab/Ad columns. When letters overlap, they are slightly offset along the horizontal axis. (B, C) Ankle motion over the wingbeat cycle, for a single wingbeat from each individual. Wingbeats were selected to convey the full range of kinematic variation. The orange circle indicates the left hip location in the insets, which provide expanded views. (B) Lateral view from late upstroke, (C) posterior view from early downstroke.

The path of motion traced by the ankle during a wingbeat cycle varied considerably across the 25 wingbeats analyzed (Fig. 4B-4C). Viewed from behind the bat, ankle motion projected on the transverse (yz-) plane is a figure-8 pattern for 19 of 25 wingbeats and an elliptical pattern for the remaining six wingbeats. Moreover, different starting and stopping locations were not uncommon (Fig. 4C). The variation in these motion patterns indicated that the mediolateral and dorsoventral motion are not entirely coupled.

The amplitude of ankle motion showed a similar high level of variation. Flexion/extension amplitude ranged from 23° to 73°, and was on average 2.4 times greater than abduction/adduction amplitude, which ranged from 6° to 34° (Fig. 4A). The position of the ankle was dorsal to the hip and shoulder markers for 84±3% (mean ± s.e.m.) of the wingbeat duration, and lateral to the hip and shoulder markers for 96±2% of the wingbeat duration.

**Figure 5:**
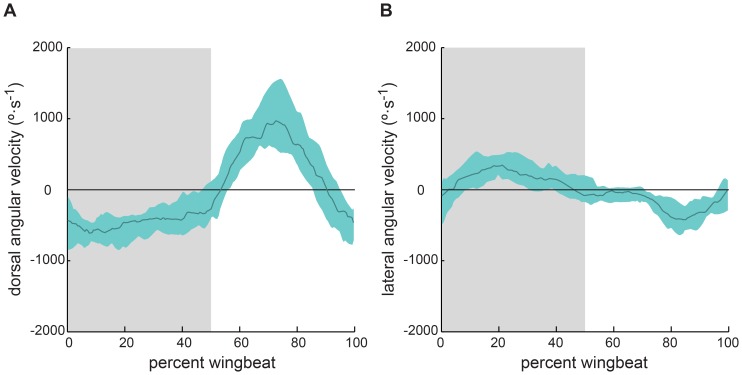
Direction of ankle motion was consistent among wingbeats. Angular velocity of the ankle with respect to the hip in (A) the parasagittal (xz-) plane, with positive indicating dorsal extension, and (B) the frontal (xy-) plane, with positive indicating abduction. Solid line is median; aqua shaded envelope is bounded by the first and third quartiles over *N* = 5 individuals, 25 wingbeat cycles. Downstroke is shaded in gray.

Despite variability in ankle position during the wingbeat cycle, there was a clear relationship between the direction of ankle motion and phase of the wingbeat cycle. The ankle moved ventrally throughout the entire downstroke and began moving dorsally almost synchronously with commencement of the upstroke. However, the ankle moved ventrally before the commencement of downstroke. Thus, ventral motion of the ankle occured over a longer portion of the wingbeat cycle than that of the wrist, while ankle dorsal motion occured rapidly, over a shorter period (Fig. 5A). In the mediolateral direction, the ankle tended to move laterally during downstroke and medially during upstroke (Fig. 5B).

### Trailing edge kinematics

**Figure 6:**
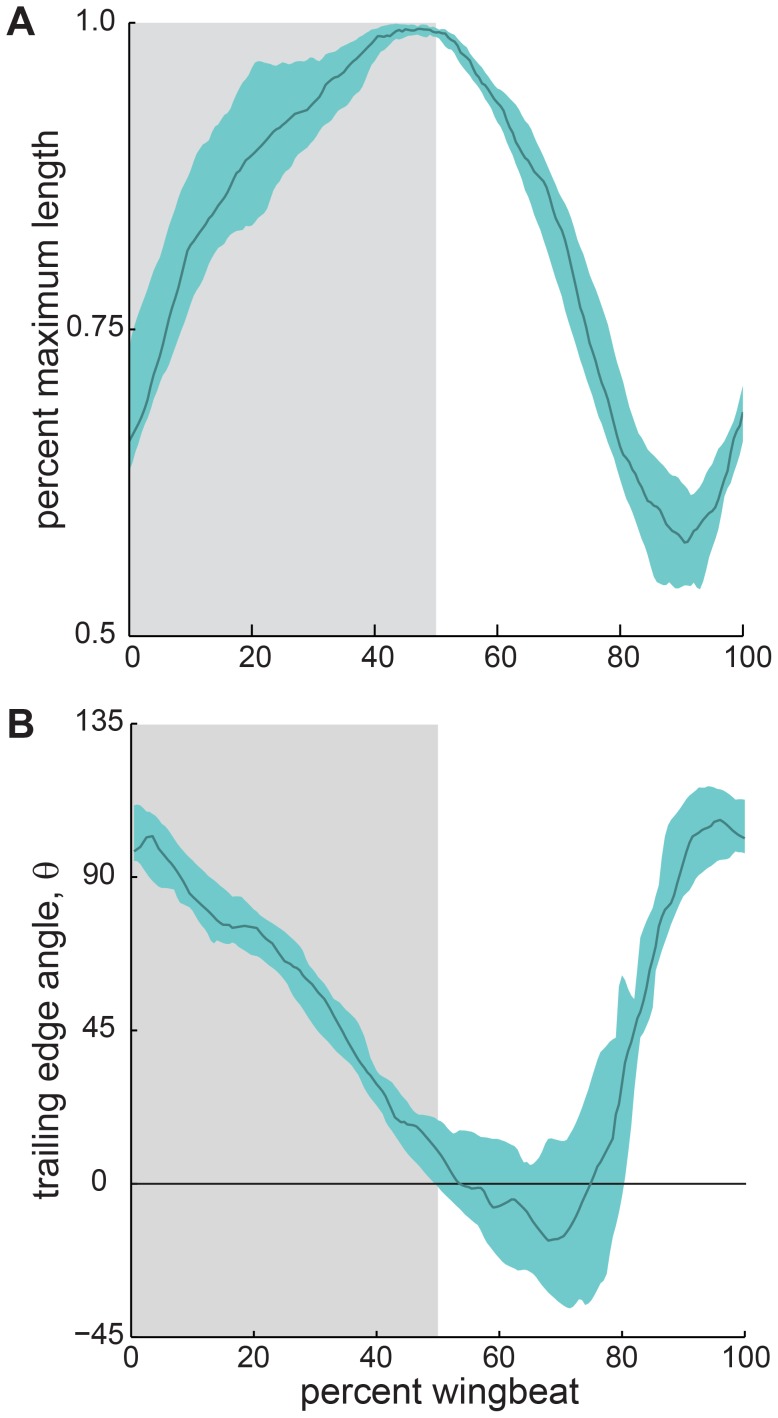
Wing membrane tension varied in a consistent pattern among wingbeats. Proxies for magnitude and orientation of forcing of the ankle by the wing membrane through the wingbeat cycle. (A) Length of the trailing edge normalized by maximum length, a proxy for the magnitude of force. (B) Projected on the transverse (yz-) plane, angle of the trailing edge at the ankle relative to the y-axis (θ), a proxy for the direction of force. Solid line is median; aqua shaded envelope is bounded by the first and third quartiles over *N* = 4 individuals, 17 wingbeat cycles. Downstroke is shaded in gray.

We found a clear relationship between wingbeat cycle timing and trailing edge length (Fig. 6A). The wing membrane lengthened quickly from ca. 65% to 85% of its maximum length over the first 15% of the wingbeat and then lengthened gradually to its maximum length over the remainder of the downstroke. Maximum length was reached and maintained during the downstroke to upstroke transition, between 45% and 55% of the wingbeat duration. The trailing edge then rapidly shortened to ca. 55% of its maximum length between 55% and 90% of the wingbeat duration, at which point the wing membrane proceeded to elongate again prior to the beginning of downstroke.

The orientation of the wing membrane trailing edge at its ankle attachment (trailing edge angle, θ, in Fig. 6B) was also tightly correlated with the timing of the wingbeat cycle. At the beginning of downstroke, the trailing edge was oriented approximately dorsally (close to 90°), but as the downstroke progressed and the membrane lengthened, the orientation of the trailing edge became more lateral. During the downstroke to upstroke transition, between 45% and 55% of the wingbeat duration, the median observed trailing edge angle changed from ca. 15° to 0°. Near 70% of the wingbeat duration, the angle of the trailing edge rapidly reoriented dorsally, and it began to decrease again at 90% of the wingbeat duration.

### Interactions between wing membrane tension and ankle motion

The dorsoventral components (z) of wing membrane tension and ankle velocity were opposed throughout 71±2% of the wingbeat. During these periods, the wing membrane removed power from ankle motion. Considering downstroke and upstroke separately, ventral ankle velocity opposed wing membrane tension for 91±3% (mean ± s.e.m.) of downstroke duration and was only aligned during late downstroke. In contrast, these two vectors were opposed for 48±5% of upstroke duration and were most commonly aligned during the middle third of upstroke.

## Discussion

**Figure 7:**
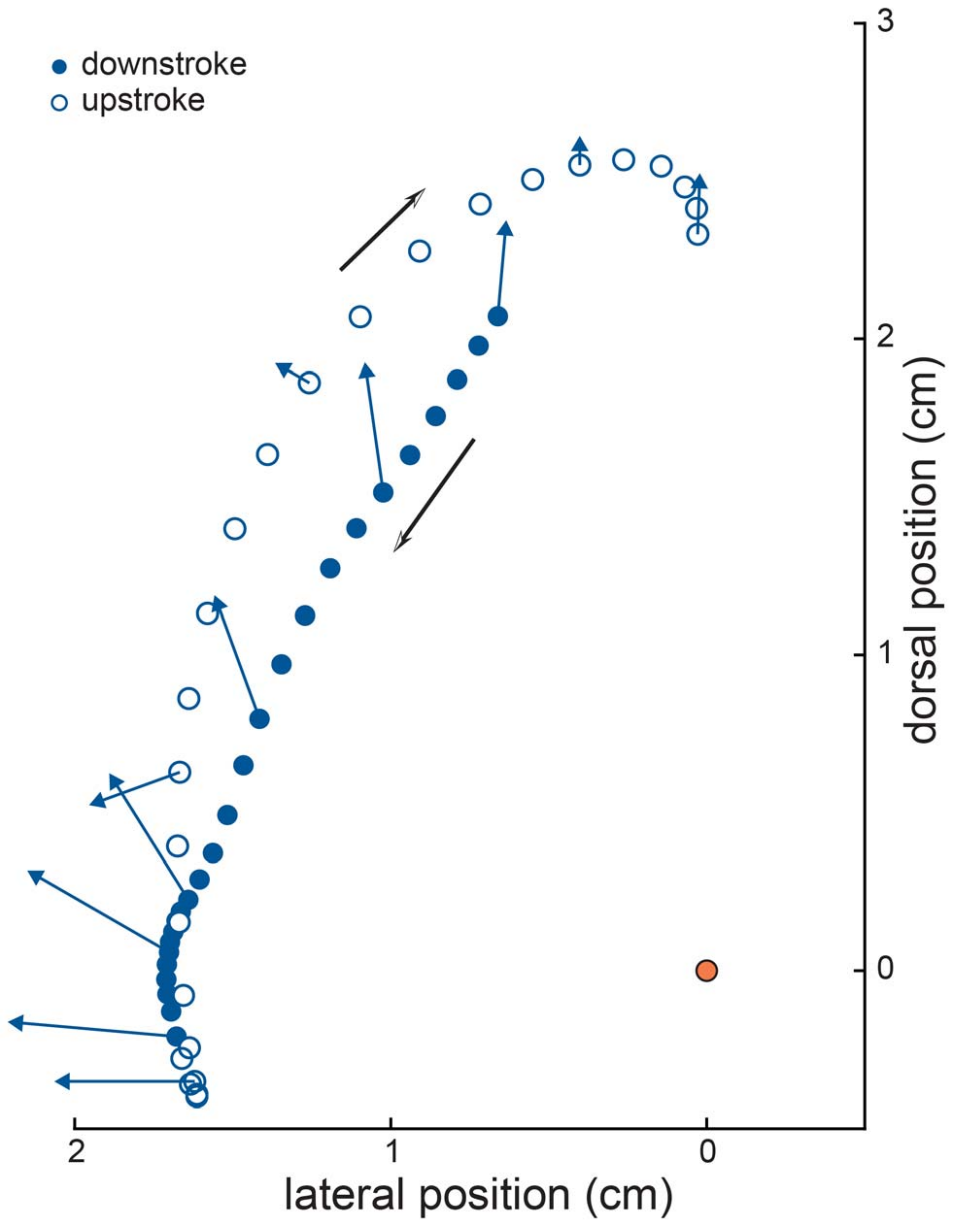
Ankle motion consistently resisted wing membrane tension. Left ankle motion and approximate wing membrane tension through one wingbeat cycle in the transverse (yz-) plane (view as in Fig. 3B). Filled markers indicate movement during downstroke; open markers indicate movement during upstroke. Blue arrows indicate direction and approximate magnitude of wing membrane tension on the ankle, at 10% intervals across the wingbeat cycle. Black arrows indicate direction of ankle motion. Orange circle indicates location of left hip. Data is from individual 4, wingbeat β.

The physical attachment of the hindlimb to the wing membrane allows it to influence wing shape. Hindlimb position affects both the chord- and spanwise shape of the wing, and in turn, three-dimensional wing conformation strongly influences the lift, drag, and pitching moment that the bat experiences. We found that the ankle moved roughly in phase with the wingbeat cycle: during downstroke, the ankle moved ventrally and laterally, and during the majority of upstroke, the ankle moved dorsally and medially, with dorsoventral motion on average being 2.4 times greater than mediolateral motion (Fig. 4; Fig. 5). The dorsoventral component of ankle motion opposed wing membrane tension with a high degree of consistency during downstroke (Fig. 7). This result is not consistent with the hypothesis that passive wing membrane tension is sufficient to explain the observed hindlimb motion. Therefore, we propose that hindlimb muscle activity and/or inertial effects are responsible for powering hindlimb movements. Whatever the proximate cause, ventral ankle motion during downstroke increases wing angle of attack and modifies wing shape. This motion is accompanied by flexion at the hip and/or knee, which adjust the overall wing profile as well as lift, drag, and pitch produced by the wing.

### Ankle marker kinematics

Few studies have examined hindlimb movements in bat flight (but see [7], [9], [10]). Our observations extend these studies by describing hindlimb movements with improved spatial and temporal resolution, as well as increasing the number of observations. Our results confirm some previous observations. In *C. brachyotis*, the foot swept through a large arc in the parasagittal (xz-) plane (Fig. 4B), as previously observed in *Glossophaga soricina,* a small-bodied nectar-feeding phyllostomid [7]. We also found substantial variation in the magnitude of ankle motion among wingbeats, as previously observed in several bat species from diverse families (Fig. 4A) (see Fig. 2 in [9]; Fig. 2 in [10]). However, despite little consistency among wingbeats between ankle position and wingbeat cycle timing, there is a relationship between dorsoventral trajectory of ankle motion and wingbeat cycle timing that is consistent among wingbeats and individuals (Fig. 5).

### Interactions between wing membrane tension and ankle motion

Ankle motion was generally opposed by tension exerted by the wing membrane. During downstroke, the dorsoventral component of force that the wing membrane exerted on the ankle was directed dorsally (θ>0 in Fig. 6B), yet the ankle moved ventrally (Fig. 5A). In a few trials, the wing membrane exerted force in the ventral direction at the end of downstroke, but the ventral component of force was probably very small because the wing membrane pulled in an almost purely lateral direction during this time (Fig. 6B).

Ankle motion was potentially aided by wing membrane tension during approximately half of the upstroke duration. However, during this portion of the wingbeat cycle, the wing membrane was either taut and pulled primarily laterally (see first half of upstroke in Fig. 6), or relatively slack and pulled dorsally (see second half of upstroke in Fig. 6). This pattern of movement arose from the relative timing of wing membrane shortening and wing membrane reorientation. During downstroke, the wing membrane elongated under tension, as aerodynamic loading increased. Then, at the initiation of upstroke, the wing membrane rapidly shortened, and throughout this period, orientation of wing membrane tension at the ankle was primarily lateral. As the wing membrane approached minimum length, the orientation of tension on the ankle rapidly reoriented dorsally. Hence, although wing membrane tension was aligned with ankle motion during a significant period of the upstroke, the magnitude of the dorsally-oriented force exerted by the membrane on the ankle was low (Fig. 7).

The interactions between wing membrane tension and ankle motion imply a primarily, if not purely, resistive role of wing membrane tension on ankle motion. For dorsoventral ankle velocity to be consistently opposed by wing membrane tension, hindlimb muscle forces and/or inertial effects must be aligned with ankle velocity. One or both of these forces powers dorsoventral hindlimb movements, and membrane tension dampens them.

### Wing membrane elongation

The changes in trailing edge length that we documented were surprisingly large (Fig. 6A). The wing membrane elongated almost 100% of the minimum length observed during a typical wingbeat cycle. This would approach or exceed the failure strain observed in many studies of mechanical behavior of vertebrate skin [16]. However, bat wing membrane skin is distinguished from typical mammalian skin by the presence of large macroscopic elastin fiber bundles [17], [18]. Elastin can more than double its resting length before yielding [19], [20], and mechanical tests have revealed similarly large failure strains for bat wing membranes [16]. We therefore suggest that these measurements are realistic, and that future studies could employ additional markers to further resolve the shape and motion of the trailing edge.

### Aerodynamic consequences of hindlimb movement in bat flight

The bat hindlimb affects wing shape both by its movement dynamics and anatomy. The effects will be greater on portions of the wing close to the hindlimb and body and decrease distally, with little to no effect in the hand-wing. This gradient in influence is caused by the attachment of the wing membrane proximally to the lateral aspect of the body wall, thigh and leg, and more distally, to digit V; at these attachments, the membrane must reflect the shape of the skeletal elements to which it is affixed. Because the hindlimb defines part of the boundary conditions for the wing membrane, it restricts how the wing membrane deforms in response to aerodynamic load [21].

Varying boundary conditions significantly affects aeromechanics of wing membranes [22]-[24]. However, empirically quantifying magnitude of aerodynamic effects caused by hindlimb motion is challenging. Wing shape is a function of both the skeletal kinematics of the wingbeat cycle and aerodynamic loading. Bat wings reconfigure, and thus change the boundary conditions of the wing membrane, throughout the wingbeat cycle, which change the aeroelastic interactions with airflow [13], [21]. Attributing changes specifically to the hindlimb is impossible without detailed measurements of wing shape and aerodynamic force. However, large-scale aerodynamic consequences of hindlimb posture in bats have been explored with a physical modeling approach using a fixed, membrane-wing model with hindlimbs capable of hip flexion [5]. Angling the hindlimbs of the model relative to its body axis, in a manner that mimicked ventral movement of the hindlimb, increased lift and lift-to-drag ratio and reduced pitching moment in most configurations. Increased lift and lift-to-drag ratio were attributed to increased angle of attack and camber, while the reduced pitching moment was likely a result of limb flexion increasing wing camber and/or shifting the relative location of maximum camber toward the trailing edge [25], [26].

The chordwise location of maximum wing camber provides a reasonable estimate of the pitching moment of a thin wing section [25], [26]. In bats, wing profile, including the chordwise location of maximum camber, changes proximodistally along the wingspan. It is relatively straightforward to determine the chordwise wing profile where the wing membrane is constrained at the body and hindlimb, and along digit V. At these locations along the wingspan, maximum camber occurs at a ventral-flexing joint in the wing skeleton (Fig. 2). This will most likely be at either the hip or knee; the knee is a possibility because of the evolutionary reconfiguration of the hip, which allows the knee to move the leg and ankle to a more ventral and cranial position (Fig. 1). Thus, the anatomical orientation of the hip joint in bats and the movement dynamics of the hindlimb are important in determining the three-dimensional configuration of the wing, and hence in determining the aerodynamics of bat flight.

## Conclusions

Because the wing membrane is attached to the hindlimb, bats can change wing shape by repositioning the hindlimbs. We found considerable variability in ankle motion among wingbeats, yet the pattern of ankle motion was highly predictable over wingbeat cycles as a whole. The temporal patterns of ankle motion, length-changes of the wing membrane trailing edge, and orientation of the trailing edge relative to the ankle led us to reject the hypothesis that the motion of the hindlimb arises passively through tension in the wing membrane. We therefore propose that hindlimb motion is generated directly by muscle activity and/or inertial effects from vertical body oscillations and is resisted by membrane tension. Further study is needed to distinguish among these alternatives. Irrespective of the proximate cause, the observed hindlimb movement must significantly influence three-dimensional wing conformation, and thus affect aerodynamics in complex ways.

## Supporting Information

File S1 Supplemental material.
(XLSX)
